# Relevance of NLRP3 Inflammasome-Related Pathways in the Pathology of Diabetic Wound Healing and Possible Therapeutic Targets

**DOI:** 10.1155/2022/9687925

**Published:** 2022-06-30

**Authors:** Youjun Ding, Xiaofeng Ding, Hao Zhang, Shiyan Li, Ping Yang, Qian Tan

**Affiliations:** ^1^Department of Burns and Plastic Surgery, Nanjing Drum Tower Hospital Clinical College of Jiangsu University, No. 321, Zhongshan Road, Nanjing, Jiangsu, China; ^2^Department of Emergency Surgery, The Fourth Affiliated Hospital of Jiangsu University (Zhenjiang Fourth People's Hospital), 20 Zhengdong Road, Zhenjiang, Jiangsu 212001, China; ^3^Department of Burns and Plastic Surgery, Nanjing Drum Tower Hospital Clinical College of Traditional Chinese and Western Medicine, Nanjing University of Chinese Medicine, No. 321, Zhongshan Road, Nanjing, Jiangsu, China; ^4^Department of Burns and Plastic Surgery, Nanjing Drum Tower Hospital, The Affiliated Hospital of Nanjing University Medical School, No. 321, Zhongshan Road, Nanjing, Jiangsu, China; ^5^Department of Burns and Plastic Surgery, Anqing Shihua Hospital of Nanjing Drum Tower Hospital Group, Anqing, Anhui, China

## Abstract

Wound healing is a major secondary complication in type 2 diabetes, which results in significant disability and mortality, imposing a significant clinical and social burden. Sustained activation of the Nod-like receptor protein (NLRP) inflammasome in wounds is responsible for excessive inflammatory responses and aggravates wound damage. The activation of the NLRP3 inflammasome is regulated by a two-step process: the priming/licensing (signal 1) step involved in transcription and posttranslation and the protein complex assembly (signal 2) step triggered by danger molecules. This review focuses on the advances made in understanding the pathophysiological mechanisms underlying wound healing in the diabetic microenvironment. Simultaneously, this review summarizes the molecular mechanisms of the main regulatory pathways associated with signal 1 and signal 2, which trigger the NLRP3 inflammasome complex assembly in the development of diabetic wounds (DW). Activation of the NLRP3 inflammasome-related pathway, involving the disturbance in Nrf2 and the NF-*κ*B/NLRP3 inflammasome, TLR receptor-mediated activation of the NF-*κ*B/NLRP3 inflammasome, and various stimuli inducing NLRP3 inflammasome assembly play a pivotal role in DW healing. Furthermore, therapeutics targeting the NLRP3 inflammasome-related pathways may promote angiogenesis, reprogram immune cells, and improve DW healing.

## 1. Introduction

Diabetes mellitus (DM), the most frequently diagnosed chronic metabolic disorder, affects more than 460 million individuals worldwide, and its prevalence is projected to increase to 642 million cases by 2040 [1]. Diabetic wounds (DW) are a major secondary complication of type 2 diabetes; the development of such wounds results in an increased risk of limb amputation and disability in patients [2]. Nonhealing wounds in patients with DM negatively affect the quality of life, mortality, and morbidity, and hence, pose a critical clinical challenge and impose an economic burden worldwide [3]. In the past decade, many studies have concentrated on unresolved inflammation in the pathophysiology of DW healing, and relevant pathways have been disrupted in diabetes.

Inflammasomes, a class of cytosolic multiproteins, serve as a scaffold for caspase-dependent activation to promote cytokine maturation and the release of proinflammatory cytokines, which trigger potent inflammatory responses. In response to invading pathogens and endogenous danger signals, inflammasomes act as a part of the innate immune system against infections and mediate adaptive immune responses. Canonical inflammasomes are activated by pattern recognition receptors (PRRs), which recognize damage-associated molecular patterns (DAMPs) and pathogen-associated molecular patterns (PAMPs) [4]. The Nod-like receptor protein 3 (NLRP3) inflammasome, belonging to a class of PRRs, consists of the sensor receptor NLRP3, which refers to the nucleotide-binding oligomerization domain (NOD)-, leucine-rich repeat (LRR)-, and pyrin domain- (PYD-) containing protein 3; the adaptor protein of apoptosis-associated speck-like protein contains a caspase recruitment domain (ASC) and the effector protease caspase-1 [5]. The NLRP3-ASC-caspase-1-IL-I*β*-IL-18 axis plays a role in DW pathophysiology by regulating necroinflammation [6]. This review focuses on the molecular mechanisms underlying the NLRP3 inflammasome activity involved in the priming/licensing (signal 1) step and the protein complex assembly (signal 2) step in the pathogenesis of DW. It also summarizes the therapeutic approaches targeting the NLRP3 inflammasome and its related signals.

## 2. Introduction to NLRP3 Inflammasome

**Figure 1:**
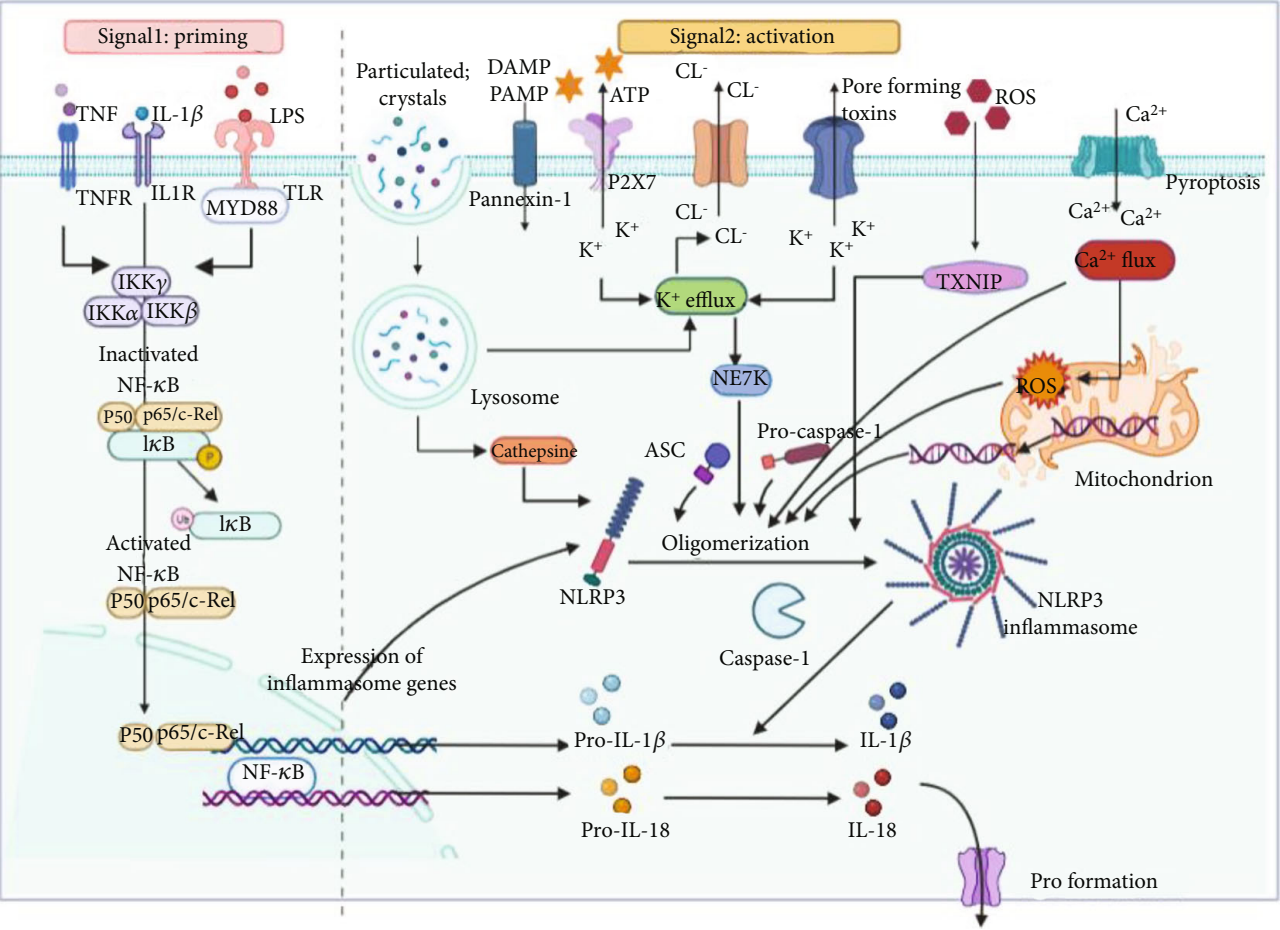
Overview of the molecular mechanisms of the priming and activation of the NLRP3 inflammasome. Activation of NLRP3 inflammasome involves a two-step process. The signal 1 (left) is referred to as priming that is primarily regulated by NF-*κ*B-dependent transcriptional of NLRP3 components, which is activated by the stimuli of TLRs, TNFs, and cytokine receptors. The signal 2 (right) of NLRP3 inflammasome activation is induced by PAMPs and DAMPs. In addition, numerous molecular or cellular events include ROS generation, ion flux, mitochondrial dysfunction, and lysosomal destabilization that trigger the oligomerization of NLRP3, ASC, and procaspase-1 into NLRP3 inflammasome complex, which lead to the cleavage of procaspase-1 into caspase-1. The activated caspase-1 cleaves the pro-IL-1*β* and pro-IL-18 into active forms IL-1*β* and IL-18. Abbreviation: PAMPs: pathogen-associated molecular patterns; DAMPs: damage-associated molecular patterns; NLRP3: Nod-like receptor protein; ROS: reactive oxygen species; TLRs: Toll-like receptors; TNFs: tumor necrosis factor; NF-*κ*B: nuclear factor kappa-B; TXNIP: thioredoxin-interacting protein; LPS: lipopolysaccharide.

A two-step process is involved in the activation of NLRP3 inflammasomes [7] (Figure 1). Signal 1 corresponds to priming and licensing and is accompanied by priming during the transcription and posttranslation events of the NLRP3 inflammasome components. The licensing of the NLRP3 protein sensor plays a vital role in this process. Signal 2, which activates the NLRP3 inflammasome, involves exogenous pathogens and endogenous damage molecules and promotes the assembly of NLRP3, ASC, and procaspase-1 into a protein complex, which subsequently releases proinflammatory cytokines [8, 9].

### 2.1. Signal 1: Priming and Licensing

It is generally accepted that the process of priming and licensing involves the transcriptional and posttranslational activation of NLRP3, pro-IL-18, and pro-IL-1*β* along with the license for rapid activation towards the assembly of the NLRP3 inflammasome complex [10, 11]. Accumulating evidence suggests that nuclear factor kappa-B (NF-*κ*B) signaling and the Fas-associated protein with death domain- (FADD-) caspase-8 pathway play essential roles in the priming response to proinflammatory cytokines and PRRs [12, 13]. NF-*κ*B signaling can be activated by stimulation with molecules such as lipopolysaccharide (LPS), interleukin-1*β* (IL-1*β*), and tumor necrosis factor- (TNF-) *α* via upstream LPS/Toll-like receptor 4 (TLR4), IL-1*β*/IL-1R1, and TNF/TNF receptor pathways, triggering the upregulation of the transcriptional expression of NLRP3 inflammatory components [14, 15]. In addition, FADD-caspase-8 could influence the priming process by acting as an upstream regulator of the NF-*κ*B signaling or triggering the activation of receptor-interacting serine/threo-nine-protein kinase (RIPK) 3/mixed lineage kinase domain-like pseudokinase (MLKL), which is required for mediating all processes [16].

### 2.2. Signal 2: Activation (Assembly) of the NLRP3 Inflammasome

The second step of NLRP3 inflammasome activation is mediated by PAMPs and DAMPs. In addition, numerous molecular or cellular events, including ROS generation, ion flux, mitochondrial dysfunction, and lysosomal destabilization, trigger the oligomerization of NLRP3, ASC, and procaspase-1 into the NLRP3 inflammasome complex, which leads to the cleavage of procaspase-1 into caspase-1. Activated caspase-1 cleaves pro-IL-1*β* and pro-IL-18 into active forms IL-1*β* and IL-18. The K+ efflux induced by various PAMPs/DAMPs can trigger the activation of NLRP3 inflammasome via pannexin-1. Intracellular hypokalemia caused by a decrease in cytosolic K+ is sufficient to activate the NLRP3 inflammasome [17]. In addition, since they act as K+ efflux channels, tandem pore domains in the weak inward rectifying K+ channel 2 (TWIK2) and P2X7 receptor (P2X7R) are necessary for this process [18, 19]. Similarly, an increase in intracellular Ca2+ concentration induced by endoplasmic reticulum (ER) Ca2+ release can also activate the assembly of the NLRP3 inflammasome through thioredoxin-interacting protein (TXNIP) [20, 21]. Furthermore, the exogenous and endogenous stimuli could induce the generation of mitochondrial reactive oxygen species (mtROS) by activating oxidative systems and thereby promote assembly and activation of NLRP3 inflammasome. Under oxidative stress conditions, nuclear factor erythroid-2-related factor (Nrf2) and TXNIP play vital roles [22, 23].

## 3. Main Factors Involved in the Pathogenesis of Diabetic Wound

Wound healing refers to the repair of damaged tissues and organs in the human body. In contrast to normal wound healing, which displays definite and orderly three phases, including inflammation, proliferation, and remodeling [24], the healing process of DW is impaired. It is associated with deficient angiogenesis, excessive formation of neutrophil extracellular traps (NETs), and phenotypic imbalance in macrophages (M*φ*), which results in a persistent inflammation that aggravates the impaired DW healing.

### 3.1. Altered Angiogenesis in DW

Angiogenesis refers to the formation of new capillaries from an established vasculature [25]. One hallmark in the wound healing is robust angiogenesis [26]. The interplay between fibroblasts and endothelial cells (ECs) is centered on the role of numerous growth factors in both physiological and pathological angiogenesis [27, 28]. Vascular endothelial growth factor (VEGF) expressed by fibroblasts is essential mediators for angiogenesis and collagen synthesis that stimulate vessel formation in ECs [27], whereas VEGF that functions as proangiogenic factor was remarkably reduced in patients with diabetic foot ulcer (DFU) and diabetic models [29]. The reduction in the expression of proangiogenic factors is responsible for the dysfunction of ECs, which results in poor EC growth, proliferation, and then impairing angiogenesis and wound healing [30]. NLRP3 inflammasome-related inflammation mediators are closely connected with the process of abnormal angiogenesis in wound healing. The effects of VEGF depend on its receptors that VEGF receptor-1 causes inflammation but VEGF receptor-2 leads to angiogenesis [31]. As discussed in further detail below, controlling NLRP3 inflammasome is considered as a useful treatment in DW, which could be evidenced by the increased expressions of angiogenesis-related factors, including VEGF and platelet-derived growth factor (PDGF) [32]. Hypoxia attributed to increased blood viscosity is also a key activator of the development and progression of abnormal vascular growth [33]. HIF-1*α*, as a critical regulator of oxygen homeostasis, plays a determinant role in healing outcomes [34]. In wounds, HIF-1 as a transcription activator regulates downstream target gene VEGF to responsible for angiogenic events that are altered in diabetes [35]. Exposure to hyperglycemia, the downregulation of HIF-1*α* causes low expression of VEGF and has a negative impact on ECs, which are susceptible to apoptosis and increased detachment and account for poor healing in DM patients [30, 36, 37]. Meanwhile, treatment with HIF-1*α* stabilizer could stimulate angiogenesis by activating HIF-1*α*/VEGF pathway to accelerate DW healing [32]. Pharmacological intervention that elevated expression of HIF-1*α*/VEGF pathway could further reduce the levels of inflammatory factors (IL-1*β*, TNF-*α*, and IL-6) [28, 38]. Furthermore, vascular endothelial injury induced by inflammation factors can lead to abnormal vascular proliferation, luminal narrowing, and poor local blood flow, which aggravates local hypoxia, thereby forms a vicious cycle, hindering DW healing [26, 39]. Overall, the multiple changes in proangiogenic and vascular maturation factors in diabetes are influenced by proinflammatory environment in DW, which perturb the proper wound healing with a large array of insufficient angiogenesis [40].

### 3.2. Role of NETs in DW

Neutrophils, regarded as a double-edged sword, are the first immune cells to migrate into the wound bed and participate in the elimination of pathogens and damaged cells. Inflammation is a key step required for initiation of wound repair. While a suitable infiltration of neutrophils in the wound allows for prevention of infection, prolonged persistence of neutrophils drives tissue damage in which injured tissues and the inflammatory response self-sustain each other [41]. In response to pathogen invasion, neutrophils release modified chromatin structures, such as their nuclear and granular contents, to form a meshwork known as NETs. Abnormal activation of NET can also contribute to the inflammatory loop [42]. NETs persist in diabetes, and their continuous presence can predisposed patients to mortality in the form of NETosis, which has been confirmed in clinical trials in patients with DFU. Furthermore, the initiation of NETosis is closely related to peptidylarginine deiminase 4 (PAD4), whose expression is upregulated in DW [43]. Inhibition of NETosis by Cl-amidine (PAD4 inhibitor) and disruption of NETs by DNase 1 can ameliorate chronic inflammation in wounds and is conducive to wound healing [44]. In addition to its regulation of macrophages through the NLRP3 inflammasome to sustain a local persistent inflammatory response, NET can affect EC activity by increasing its death and motility [45, 46]. Notably, the Janus face of NET during wound healing should be considered. Recently, patients with DW who showed NETs with a low level of cathelicidin (LL-37) demonstrated poor antimicrobial action to increase the risk of wound healing-related disorders. Additionally, treatment with the macrolide clarithromycin can restore the antibacterial activities in patients via upregulation of the expression of LL-37 on NETs [47]. These studies suggest that the NET process is highly resolutive, and therapeutic tools restoring their balance with immune cells in DW are promising.

### 3.3. Focus on Macrophage Polarization in DW

M*φ* derived from monocytes participates in each phase of the wound healing process because they kill pathogenic microbes, remove damaged tissue, and produce growth factors [48]. In response to complex microenvironments and molecular mediators, M*φ* is polarized into the classical pr-inflammatory M1 or alternative anti-inflammatory M2 [49]. The orderly transition from M1 to M2 results in beneficial effects on wound healing, particularly in tissue remodeling and angiogenesis [50]. During the normal wound healing process, M1 predominates for the first three days. Subsequently, transition to the M2 phenotype is observed, which peaks on the seventh day [51]. In the DW state, chronic low-grade inflammation that persists indefinitely is a key cause of nonhealing of wounds [52], wherein M1 persists continuously and cannot transit to M2 phenotypes. In addition, Ganesh et al. [53] found that the levels of proinflammatory cytokines, including interleukin-1 beta (IL-1*β*), tumor necrosis factor-alpha (TNF-*α*), and interferon-gamma (IFN-*γ*), are elevated in DW, which induces local hyperglycemia, accumulation of advanced glycation end products (AGEs), and ROS, along with an increase in chronic inflammation [49]. The same outcome has been observed in animal experiments, in which macrophages from normal mice switched from a pro- to anti-inflammatory phenotype on the tenth day, while diabetic mice displayed a twofold higher expression of proinflammatory factors IL-1*β* and IFN-*γ* [54], which facilitated the maintenance of the proinflammatory M1 phenotype. The phenotypic imbalance of M*φ* is closely related to the activity of the NLRP3 inflammasome, which is influenced by the abundance of proinflammatory factors [55]. Importantly, the activation of the NLRP3 inflammasome in M1 with increased IL-1*β* levels can form a positive feedback loop to amplify the proinflammatory effect of M1, which is attributed to the failure of the transition of M*φ* to the M2 phenotype in DW [56]. Due to the influence of intricate etiologies in DW, the transformation from proinflammatory M1 to anti-inflammatory M2 can be deferred or even absent; this hampers the switch from the inflammatory to the proliferation phase during tissue repair, consequently impairing the wound healing process [57].

## 4. Correlation of the NLRP3 Inflammasome Pathway with DW

### 4.1. Crosstalk between Nrf2 and NF-*κ*B/NLRP3 Inflammasome (Signal 1) in DW

Nrf2, a master regulator of antioxidant defenses, modulates the redox homeostasis of cells by binding to antioxidant response elements (AREs) of antioxidative genes encoding antioxidant enzymes after heterodimerization with musculoaponeurotic fibrosarcoma oncogene homolog (Maf) proteins under endogenous and exogenous stress conditions [58]. Under physiological conditions, Nrf2 binds to Keap1, which negatively regulates Nrf2 by targeting its proteasomal degradation. However, under stress conditions, Nrf2 dissociates from the Nrf2-Keap1 complex, translocates into the nucleus, and subsequently induces the expression of antioxidant genes [59]. Nrf2 can limit the inflammatory response induced by NF-*κ*B, thereby suppressing NLRP3 inflammasome activity [60, 61]. In contrast, NF-*κ*B can reduce the expression of Nrf2 signaling by competing with Nrf2 for the transcriptional coactivator CREB-binding protein (CBP) p300 complex [62]. Furthermore, P65 (the canonical NF-*κ*B subunit) can prevent the formation of the Nrf2-Maf heterodimer by improving the recruitment of histone deacetylase 3 (HDAC3) to MafK, which decreases the expression of Nrf2-mediated ARE-related genes [63]. Therefore, the roles of Nrf2 and the NF-*κ*B/NLRP3 inflammasome in wound healing are reciprocal.

The mechanism underlying impairments in DW healing involves the induction of premature senescence in endothelial progenitor cells (EPCs) due to excessively high ROS levels in a hyperglycemic environment; this results in the deficiency of repair and angiogenesis [64]. Given that Nrf2 plays an essential role in normal endothelial angiogenesis, disruption of Nrf2 signaling might be a mechanism underlying impaired angiogenesis observed in DW healing [65]. The circulatory levels of Nrf2 and its downstream targets are reduced in patients with type 2 diabetes and DFU compared to those in control subjects [66, 67]. Similar results were also observed in diabetic rat models [68]. High glucose (HG) levels increase the levels of the p65 canonical NF-*κ*B subunit, leading to activation of the inflammatory process, which in turn, negatively regulates Nrf2 [69]. However, as an antioxidant protective mechanism, perilesional skin tissues of patients with diabetes are under the influence of high oxidative stress, which induces compensatory activation of the Nrf2, downstream heme oxygenase 1 (HO-1), and NAD(P)H quinone dehydrogenase 1 (NQO1) genes, albeit not to normal levels [70]. The pharmacological activation of Nrf2 and inhibition of NF-*κ*B promotes DW healing, which is a promising therapeutic target [71].

### 4.2. TLR Receptor-Mediated Activation of NF-*κ*B/NLRP3 Inflammasome (Signal 1) in DW

TLR signals, particularly TLR4, are essential stimuli that activate the NF-*κ*B pathway by binding to its downstream partner, MyD88 [72]. The role of TLR is primarily influenced by their activation with ligands and is closely associated with the microenvironment in DW. The expression of TLR 2, 4, and 6 was persistently upregulated from injury to the tenth day in DW, while in non-DW, the levels of TLRs recovered, reaching those observed at the baseline [73, 74]. The NF-*κ*B pathway, mediated by continuous stimuli of TLRs, contributes to diabetes-associated delayed wound healing and eventually triggers a cascade of amplified inflammatory responses that hinder wound healing. Similar findings were revealed in patients with DW; the increased TLR4 expression was observed along with that of the downstream markers of activation, including MyD88, IRAK, NF-*κ*B, and IL-1*β* [75]. Furthermore, genetic deletion of TLR4 in diabetic mice ameliorated wound healing [76]. Taken together, these studies suggest that TLR-MyD88-NF-*κ*B signaling maintains DW in an inflammatory state to delay wound healing. Notably, the NF-*κ*B pathway is the best-known signal for transcriptional activation of NLRP3 inflammasome components.

Neutrophils are the main leukocytes at wound sites and play a vital role in healing [77]. The pathogenesis of diabetes involves inflammatory or metabolic processes, such as neutrophilia and hyperglycemia, which result in the formation of NETosis mediated by neutrophils [44]. The effect of high concentrations of NETs on DW is responsible for infection and the subsequent worsening of wounds [78]. The major components of NET, including histones and DNA, are recognized by TLR receptors as endogenous DAMPs [79]. NET is a key scaffold in DW that acts as a priming signal for NLRP3 activation through the TLR4/9/NF-*κ*B pathway in macrophages. NET-driven NLRP3 inflammasome activity in M*φ* further induces the infiltration of innate immune cells into the diabetic wound, which severely impairs wound healing [80, 81].

NLRP3 inflammasome activity is required for keratinocytes to respond to a range of irritants. However, excess inflammasome/IL-1 activity in keratinocytes other than macrophages also aggravates DW healing [82, 83]. Upon exposure to HG, the NLRP3 inflammasome in keratinocytes is activated by excess ROS, which inhibits the proliferation and migration of keratinocytes. Recently, Lee et al. [84] used a model of wound healing with epidermal deletion of caspase-8 and observed an elevated level of caspase-1 mediated by NF-*κ*B, which directly binds to the caspase-1 promoter. However, the deletion of NF-*κ*B, loss of caspase-1, or inhibition of IL-1R in keratinocytes disrupted the inflammatory phase of cutaneous wound closure and impaired the proliferative phase of ECs, resulting in delayed cutaneous wound closure. Thus, basal immune response and inflammation resolution are essential homeostatic mechanisms for maintenance of wound healing.

### 4.3. Relationship between DW and NLRP3 Inflammasome Assembly (Signal 2)

When induced by PAMPs or DAMPs, the conformation of NLRP3 is changed, and the NACHT domain is exposed, which triggers oligomerization of the NLRP3 inflammasome complex (signal 2). This complex is involved in a series of signaling pathways [85]. In DW, multiple stimuli that disturb intracellular homeostasis can result in persistent NLRP3 inflammasome activity, promote maturation and secretion of proinflammatory cytokines, and hinder wound healing [86].

#### 4.3.1. Reactive Oxygen Species and Mitochondrial Reactive Oxygen Species

In addition to the stimulant role of the NLRP3 inflammasome in macrophages, the ROS-mediated pathway is required as a common step for the activation of the NLRP3 inflammasome (signal 2). The high accumulation of ROS results in an increase in membrane permeability, disrupts the balance of ions in cells, and further intensifies the stimulation of DAMPs [87]. As a key modulator of intracellular ROS, TXNIP is a pivotal mediator of ROS-induced NLRP3 inflammasome activity [88]. In the HG-induced state, the level of TXNIP protein increases, which further stimulates the downstream NLRP3 inflammasome [89]. The activity of xanthine oxidoreductase (XOR), a source of ROS in chronic wounds, is elevated, which drives ROS overproduction in the diabetic wound environment [90]. The secretion of IL-1*β*, which further amplifies inflammation, can be stimulated by XOR. Moreover, wound tissue isolated from diabetic patients shows increased expression of the NLRP3 inflammasome, which is activated by ROS in the DW environment [91]. Excess production of free radicals can cause progressive damage to DW via lipid peroxidation and protein modification, which increases their susceptibility to inflammatory responses [92]. Inhibition of ROS by N-acetylcysteine (NAC), a free radical scavenger, blocks the activation of the NLRP3 inflammasome, which accelerates impaired wound healing in a diabetic rat model [93]. Mitochondria are the main source of ROS, which act as DAMPs in damaged cells that trigger the NLRP3 inflammasome in macrophages, partially via ATP derived from mitochondria and P2X7 expression [94]. Mitochondrial DAMPs and mtROS drive DW, in which inflammation and mitochondrial dysfunction self-sustain each other. [95]. The application of mtROS scavengers on inflamed wounds attenuates IL-1*β* and IL-18 production [96]. In addition, experimental findings linking excessive ROS to the pathogenesis of diabetic wounds have been demonstrated in vitro. These findings implicate the NLRP3 inflammasome as a pathogenic mediator of downstream apoptosis. Fibroblasts derived from the wounds of patients with DFU showed an increased level of apoptosis induced by prolonged ROS-mediated activation of the NLRP3 inflammasome [97]. Apoptosis depends on the NLRP3 inflammasome as it is blocked by an inhibitor (BAY 11-7082) of the NLRP3 inflammasome. Based on these observations, the NLRP3 inflammasome is constantly activated by excessive ROS in diabetic wounds, which induces downstream inflammatory events of “glucotoxicity” [98], and thereby promotes caspase-8/3-dependent apoptosis, exacerbates skin remodeling, and impairs wound healing in diabetic patients.

**Figure 2:**
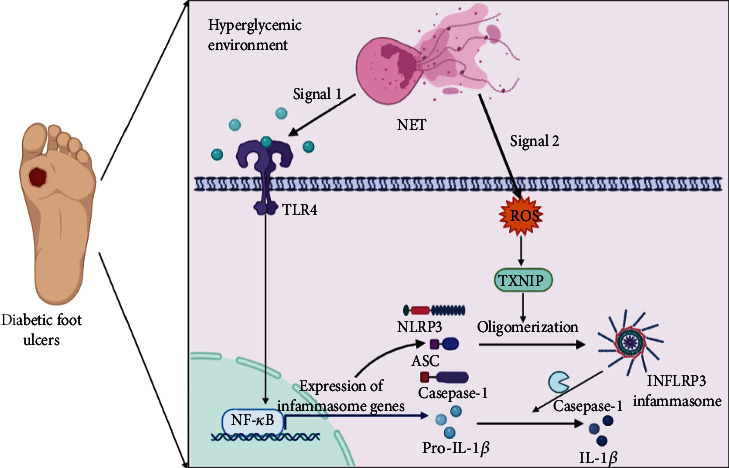
Overview of the activation of NLRP3 inflammasome in M*φ* by NETs, leading to nonresolution of inflammation in diabetic foot ulcers. DW microenvironment stimulates the neutrophils to form NETs. Stimulation of the activation of the NLRP3 inflammasome by NETs in macrophages is regulated by two steps, which involves the priming process mediated by the TLR4/NF-*κ*B pathway and the assembly process mediated by the ROS/TXNIP pathway. NET-driven NLRP3 inflammasome activity in M*φ* further induces the infiltration of the innate immune cells into the diabetic wound, which severely impairs wound healing. Abbreviation: NETs: neutrophil extracellular traps; DW: diabetic wound; M*φ*: macrophages; NLRP3: Nod-like receptor protein; ROS: reactive oxygen species; TXNIP: thioredoxin-interacting protein; TLR4: Toll-like receptor 4; NF-*κ*B: nuclear factor kappa-B.

In addition to the TLR4/9/NF-*κ*B pathway, NETs also induce the overproduction of ROS, promoting the targeting of NLRP3 by TXNIP, which stimulates the assembly of the NLRP3 inflammasome in macrophages [45] (Figure 2). Collectively, the stimulation of NLRP3 inflammasome activation mediated by NETs in macrophages is regulated in two steps: priming by the NF-*κ*B pathway and assembly by the mediated by ROS/TXNIP pathway. In addition to being driven by IL-1*β*, NETosis can also predispose to a self-perpetuating cycle of sustained IL-1*β* and IL-18 release, triggering the accumulation of proinflammatory cytokines and ROS in diabetic wound sites, which aggravates wound inflammation [99, 100]. Strategies aimed at inhibiting NETosis and eliminating NETs significantly improve the wound repair process in diabetes, uncovering a novel therapeutic approach for DWs.

#### 4.3.2. Potassium (K+) Efflux

Exogenous ATP causing K+ outflow has emerged as a key upstream event in the assembly of the NLRP3 inflammasome and release of mature IL-1*β*. P2X7R, a distinct gated ion channel, is gated by ATP to participate in the maturation of IL-1*β* via K+ efflux [101]. Macrophages from diabetic mice displayed a high level of NLRP3 inflammasome components, and treatment with zoledronate, a nitrogen-containing bisphosphonate, which augments persistent NLRP3 activation by the K+/P2X7R/ROS pathway but not by lysosomal rupture contributes to impaired oral socket wound healing [56]. In addition, evidence of the critical role of inflammasome inhibitors in DWs is centered on observations of neovascularization; the expression of such inhibitors is reduced in a diabetic environment. Persistent inflammation leads to a decrease in collagen organization and neovascularization, which is associated with several angiogenic factors, including VEGF and chemokine CXCL12 [102, 103]. For instance, drugs that block P2X7R improve angiogenesis, which is associated with the expression of VEGF and CXCL12, and ameliorate wound healing [104]. Recently, NEK7, a potassium-specific protein, was shown to activate the NLRP3 inflammasome by acting downstream of K+ efflux to trigger the process of NLRP3 oligomerization and punctate ASC aggregation, which was confirmed by the observation showing that blocking the contact between NLRP3 and NEK7 could directly inhibit the activation of the NLRP3 inflammasome [105, 106]. To understand whether NEK7/NLRP3 is associated with diabetic foot, Cai et al. [107] compared the expression of NEK7/NLRP3 between patients with and without diabetic foot to show that the NEK7/NLRP3 pathway, which was significantly increased in patients with diabetic foot, drove the pathogenesis of diabetic foot.

#### 4.3.3. Lysosomal Destabilization

Disruption of lysosomes and release of cathepsins are essential stimuli of the NLRP3 inflammasome. Phagocytosis of certain particulate matter, including cholesterol crystals, uric acid, and silica, stimulates the activation of the NLRP3 inflammasome. The phagocytosis is induced by lysosomal destabilization and rupture, followed by the release of cathepsin B into the cytoplasm [108, 109]. Cathepsin B directly connects with the C-terminal LRR domain of NLRP3, which facilitates oligomerization of the NLRP3 inflammasome [110]. Under conditions involving excess nutrition, excessive uptake of LDL particles with impaired lysosomal hydrolysis can drive lysosomal membrane permeabilization, which allows the leakage of lysosomal contents, such as lipase, cathepsins, and Ca2+, the canonical stimulators of inflammatory pathways [111]. Lipotoxicity is responsible for inflammatory dysfunction of macrophages in impaired DW healing. The connection between lipid stress and lysosomal pathology is associated with glutamine metabolism, which could overwhelm the mitochondria and further exacerbate the accumulation of saturated fatty acid (SFAs) palmitate. SFAs play a role in lysosomal dysfunction in macrophages such that they alter the responses of macrophages to inflammatory stimuli [112].

## 5. Advances in Targeting NLRP3 for the Treatment of DW

### 5.1. Therapeutic Targeting of NLRP3 Inflammasome Priming (Signal 1) in DW

**Figure 3:**
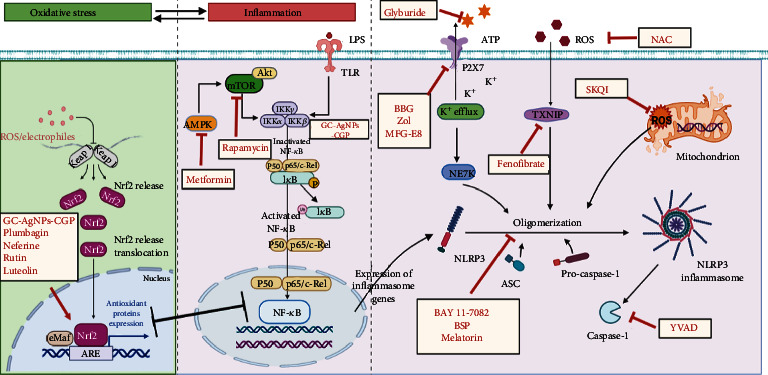
Therapeutic targeting of NLRP3 inflammasome in DW. Activation of Nrf2-mediated antioxidant defenses and suppression of NF-*κ*B/NLRP3 inflammasome-mediated anti-inflammatory action are effective therapeutic strategies, which also include GC-AgNPs-CGP, plumbagin, neferine, rutin, and luteolin. Metformin and rapamycin target AMPK and mTOR to inhibit the NF-*κ*B signaling pathway, consequently blocking the priming of the NLRP3 inflammasome. Glyburide suppresses NLRP3 oligomerization in an ATPase-dependent manner. P2X7 is a vital target of BBG, Zol, and MFG-E8. In addition, inhibition of TXNIP by fenofibrate can regulate the ROS-mediated NLRP3 inflammasome to exert a beneficial effect on DW. NAC and SKQ1 inhibit ROS and mROS, respectively, to block the assembly of the NLRP3 inflammasome. YVAD is a specific inhibitor of caspase-1. Additionally, BAY 11-7082, BSP, and melatonin directly black the oligomerization of NLRP3 to exert beneficial effect for DW healing. Abbreviation: DW: diabetic wound; NLRP3: Nod-like receptor protein; ROS: reactive oxygen species; TXNIP: thioredoxin-interacting protein; Nrf2: nuclear factor erythroid-2-related factor; GC-AgNPs-CGP: gallocatechin-silver nanoparticle-impregnated cotton gauze patches; AMPK: AMP-activated protein kinase; mTOR: mammalian target of rapamycin; BBG: brilliant blue G; Zol: zoledronate; MFG-E8: milk fat globule epidermal growth factor VIII; NAC: N-acetylcysteine; mROS: mitochondrial reactive oxygen species; BSP: Bletilla striata polysaccharide; SKQ1: 10-(6′-plastoquinonyl) decyltriphenylphosphonium; YVAD: Tyr-Val-Ala-Asp.

Various pharmacological approaches have focused on initiation events for NLRP3 inflammasome activation, which have been approved for the treatment of DW (Table 1, Figure 3). Deubiquitination of NLRP3 is required for the priming signal during NLRP3 inflammasome activation [10]. Treatment with MF-094, a selective USP30 inhibitor, can block the deubiquitination of NLRP3 and decrease the expression of its downstream target caspase-1 to promote wound healing in DFU [113]. Heparan sulfate can reduce the levels of IL-1*β*, IL-18, NLRP3, and ASC, accompanied by the increased expression of caspase-12 and proteinase inhibitor-9 that inhibit NLRP3 inflammasome assembly, thereby improving wound healing [114]. In addition, paeoniflorin could accelerate wound healing by downregulating the NF-*κ*B-mediated inflammatory response by inhibiting CXCR2 in rats with DFU [115]. In addition, common DW treatments also target the link between energy sensing and the NLRP3 inflammasome in DW. The mammalian target of rapamycin (mTOR), a threonine/serine kinase, can act as an immune regulator of anti-inflammatory and proinflammatory cytokine expression [116]. Moreover, upstream AMP-activated protein (AMPK) and downstream NF-*κ*B contribute to HG-induced inflammation. Metformin, an orally administered biguanide, improves DW healing by promoting M2 macrophage polarization via upregulation of the AMPK/mTOR signaling pathway and subsequent inhibition of the NLRP3 inflammasome [117]. The effect of rapamycin on wound healing whether delaying or promoting wound healing has been of concern [118]. Although rapamycin impairs wound healing in kidney transplantation that is associated with their antiproliferative properties [119], it is also regarded as a potential therapeutic drug for DW healing that suppresses the phosphorylation of mTOR and subsequent NF-*κ*B-mediated NLRP3 inflammasome [120]. The effects of the Wnt signaling pathway on the wound healing process could be attributed to the promotion of angiogenesis and amelioration of the inflammatory response [121]. Wnt7a can improve wound healing and inhibit autophagy and inflammation induced by HG, such as LC3A/B, p62, and NLRP3 [122].

Activation of Nrf2-mediated antioxidant defenses and suppression of NF-*κ*B/NLRP3 inflammasome-mediated anti-inflammatory effects are beneficial in diabetic wound healing. Gallocatechin-silver nanoparticle-impregnated cotton gauze patches could ameliorate DW healing by suppressing oxidative stress and inflammatory response through the Nrf2/HO-1 and TLR4/NF-*κ*B pathways, accompanied by elevated levels of antioxidant enzymes, increased levels of growth factors and Nrf2, and decreased NF-*κ*B-mediated inflammatory response [123]. It is likely that supplementation with genistein could also accelerate DW healing owing to its effects on the enhancement of Nrf2-mediated antioxidant defense, suppressing NF-*κ*B-related inflammatory responses and restoring the NLRP3 inflammasome [124]. Recently, traditional herbal medicines with many active compounds recognized as Nrf2 cofactors have been reported to be beneficial for DW healing. The active compounds (4-vinyl catechol, 4-ethyl catechol, and alkyl catechols) of *Barleria lupulina* and *Morinda citrifolia*, natural herbs, have been identified as natural Nrf2 activators, which are used in clinical DW healing. Plumbagin from *Plumbago zeylanica*, neferine from *Nelumbo nucifera*, rutin from buckwheat, and luteolin could promote diabetic wound healing by improving the expression of Nrf2-mediated antioxidant enzymes and inhibiting the expression of NF-*κ*B-associated inflammatory cytokines [125–128]. Furthermore, diet-associated compounds, such as sulforaphane and curcumin, act in a redox-sensitive manner in response to Nrf2 activation, which reduces the apoptosis of perilesional skin tissue and the expression of MMP9 to accelerate DW healing [129].

### 5.2. Therapeutic Targeting of NLRP3 Inflammasome Assembly (Signal 2) in Diabetic Wound

Recently, strategies that preferentially target NLRP3 inflammasome assembly, such as pharmacological inhibitors, have been described and are beneficial for DW repair. Reducing excessive ROS levels by the application of antioxidants that block NLRP3 inflammasome upstream signaling improves wound healing in experimental models. For example, mitochondria-targeted antioxidants, such as 10-(6-plastoquinonyl) decyltriphenylphosphonium (SkQ1), can improve the resolution of the inflammatory phase in inflamed wounds, which is accompanied by a decrease in the proportion of neutrophils and an increase in the proportion of macrophages. These effects were reliant on the reduction in IL-1*β* and IL-18 levels [130]. Similarly, the inhibition of XOR-derived ROS protects against DW healing, which is associated with NLRP3 inflammasome activity. Besides, given that TXNIP is a vital regulator of the ROS-mediated NLRP3 inflammasome, inhibiting TXNIP by fenofibrate, a PPAR*α* agonist, can target NLRP3 inflammasome activation to exert a beneficial effect during DW treatment [131].

Persistent NLRP3 activation in macrophages disrupts the transformation of the inflammatory M1 phenotype to M2 phenotype, which results in delayed healing of DW [57]. Treatment with *Bletilla striata polysaccharide*, the main active ingredient of *Bletilla striata*, promotes wound healing by suppressing the NLRP3 inflammasome, and as a result, induces a switch from the proinflammatory M1 phenotype towards the prohealing M2 phenotype [132]. The external use of glibenclamide can show similar pharmacological effects by inhibiting NLRP3 inflammasome activity and reducing the expression of inflammatory factors to downregulate the MI phenotype and upregulate the M2 phenotype [48]. In addition, inflammatory responses at the site of the wound in diabetes are increasingly amplified through the inflammatory loop involving “NLRP3 inflammasome-NETs.” Conditional deletion of milk fat globule epidermal growth factor VIII (MFG-E8) in diabetic mice aggravates wound damage and displays exaggerated activation of the NLRP3 inflammasome with largely fixed NETs. After treatment with recombinant MFG-E8, the production of NETosis is reduced, the activities of the NLRP3 inflammasome are significantly reduced, and the rate of DW healing is accelerated [100, 133]. The underlying protective mechanism of MFG-E8 is mediated via integrin *β*3 and limiting P2X7R to regulate the activation of the NET-primed NLRP3 inflammasome.

Induced by the presence of bacteria or viruses, the NLRP3 inflammasome releases active caspase-1, which cleaves GSDMD into GSDMD-NT, triggering cell membrane pore formation, cell rupture, and release of inflammatory mediators [134]. This inflammatory cascade results in a rapid programmed cell death pattern accompanied with a robust inflammatory response, called “pyroptosis,” which is also involved in the inhibition of the wound healing process in DFUs. Recently, Pastar et al. [135] showed that the process of pyroptosis was induced by intracellular *Staphylococcus aureus* in DFU tissue due to the striking suppression of perforin-2 (P-2), which dampens wound healing in patients with DFUs. Importantly, therapeutic approaches that restore P-2 levels could protect the skin against bacterial pathogens by suppressing the intracellular accumulation of *S. aureus* and targeting the pyroptotic pathway, which effectively promotes healing.

## 6. Limitations, Challenges, and Future Directions

DW displays a sustained inflammatory phase that has been identified as the most dysregulated process in wound healing, which impedes progression to the proliferative and remodeling phase [136]. Although the NLRP3 inflammasome exerts physiological roles in protecting the body against infection and producing cytokine, sustained activation triggers inflammatory cascade with “hurt both enemies and selves” [137]. Therefore, controlling it to maintain a balanced state will resolve inflammation and accelerate wound healing. With the application of multiomics approaches, transcriptomics, and proteomics of single cells, previously unidentified targets are gradually revealed to decipher the mechanisms of pathogenicity in DW healing [138]. While the healing process of DW is complex and dynamic, these different inflammation pathways also have complex connections, as the NLRP3 inflammasome just scratching the surface. At present, there still lack studies about the crosstalk between multiple signal pathways in the DW or the changes of NLRP3 inflammasome in the different phases of DW healing. Thus, further in-depth research of the other factors in the processes of the wound healing phases will also provide us novel insights to clarify the pathogenic mechanisms of DW. Besides, new therapeutic approaches involving the multitarget, novel carrier, stem cells, and combination approaches have been evaluated and applied into clinic, while how to optimize personalized strategies with improvement of chronic inflammatory state in DW should be considered.

## 7. Conclusion

NLRP3 detects danger signals, leading to the activation of the NLRP3 inflammasome and release of proinflammatory cytokines, which play a vital role in DW. Significant advances have revealed the relationship between the molecular mechanisms of the priming/assembly step of NLRP3 inflammasome activation and the pathogenesis of DW. However, the crosstalk between other signaling pathways and NLRP3 inflammasome in DW healing and the differences in the expression of NLRP3 inflammasome from the temporal and spatial aspects, which influence the healing of DW, still need to be revealed. Undoubtedly, further in-depth study of the NLRP3 inflammasome may provide a novel interpretation of the healing process in DW, and the development of inflammasome agonists will provide a better option for patients with DW.

## Figures and Tables

**Table 1:** Summary of treatment strategies by modulating NLRP3 inflammasome pathways in DW.

Signal 1	Agent	Mechanism	Indicators	Diabetic wound healing	Model
	GC-AgNPs-CGP	Activate Nrf2/HO-1 and inhibit TLR4/NF-*κ*B pathways	MDA↓, SOD↑, Nrf2↑, Nqo-1↑, HO-1↑, Keap-1↓, MMP-2↓	Improve the wound healing	CGP dressed diabetic Sprague Dawley rats
	Genistein	Improve cutaneous Nrf2-related antioxidant and reduce NF-*κ*B-associated inflammation	NLRP3↑, ASC↑, caspase-1↑, IL-1*β*↑, NF-*κ*B↓, Nrf2↑, HO-1↓	Accelerate delayed wound healing	Alloxan monohydrate induced mice
	Plumbagin	Improve antioxidant status and reduce inflammation	Nrf2↑, MMP-2↓, NF-*κ*B↓, TNF-*α*↓, IL-6↓, IL-1*β*↓	Improve wound healing activity	STZ-induced Wistar albino rats
	Neferine	Inhibit inflammatory cytokines and Nrf2 pathway	Nrf2↑, SOD↑, CAT↑, GPx↑, NF-*κ*B↓, TNF-a↓, IL-6↓, IL-1*β*↓	Promote faster wound healing	STZ-induced Wistar rats
	Rutin	Reduce oxidative stress and inflammatory response	Nrf2↑, SOD1↑, GPx↑, HO-1↑, MMP-2↓, NF*κ*B↓, TNF-*α*↓, IL-6↓, IL-1*β*↓	Promote wound healing	STZ-induced Wistar rats
	Luteolin	Inactivate NF-*κ*B and upregulate Nrf2	Nrf2↑, SOD1↑, GPx↑, MMP-9↓, NF-*κ*B↓, TNF-*α*↓, IL-6↓, IL-1*β*↓	Promote wound restoration	STZ-induced rats
	Metformin	Regulate AMPK/NLRP3 inflammasome pathway	NLRP3↓, IL-1*β*↓, caspase-1↓, p-AMPK/AMPK↑, p-mTOR/mTOR↓, IL-10↑	Accelerate the wound healing	Sprague-Dawley rats
	Rapamycin	Inhibit mTOR/NF-*κ*B pathways	NLRP3↓, ASC↓, caspase-1↓, mTOR phosphorylation↓, NF-*κ*B↓	Accelerate the wound healing	THP-1-derived macrophages
	Wnt7a	Regulate high autophagic and inflammatory response	NLRP3↓, LC3A/B↓, IL-1*β*↓, caspase-1↓, TLR4↓, p62↓, TNF-a↓	Accelerate diabetic wound healing process	STZ-induced Sprague-Dawley rats
	Paeoniflorin	Inhibit NF-*κ*B and NLRP3 signaling pathways	CXCR2↓, NF-*κ*B↓, NLRP3↓, cleaved caspase-1↓	Attenuate wound inflammation and better wound healing	STZ-induced Sprague-Dawley rats
	Topical calcitriol application	Suppress NLRP3-IL-1*β* signaling pathway	NLRP3↓, pro-IL-1*β*↓, IL-1*β*↓, cleaved caspase-1↓, ASC↓	Promotes corneal wound healing	STZ-induced C57BL/6 mice
	MF-094	Inhibit the NLRP3 inflammasome	NLRP3↓, caspase-1 p20↓	Accelerate diabetic wound healing	STZ-induced rats
	Sulforaphane	Alleviate oxidative stress, increase proliferation and migration, decrease apoptosis	Nrf2↑, HO-1↑, NQO1↑, TGF-*β*1 ↑, MMP9↓	Promote diabetic wound healing	STZ-induced diabetic mice
	Cinnamaldehyde	Alleviate oxidative stress, increase proliferation and migration, decrease apoptosis	Nrf2↑ ,HO-1↑, NQO1↑, TGF-*β*1↑, MMP10↓	Promote diabetic wound healing	STZ-induced diabetic mice

Signal 2					
	BAY 11–7, 082	Selectively inhibit NLRP3 inflammasome activity	Active caspase-1↓, IL-1*β*↓, IL-18↓, VEGF↑, and CXCL12↑	Improve the impaired healing pattern: a decrease time to complete skin healing	Db/db mice
	BBG	Purinergic P2X7 receptor inhibitor	Active caspase-1↓, IL-1*β*↓, IL-18↓, VEGF↑, and CXCL12↑	Improve the impaired healing pattern: a decreased time to complete skin healing	db/db mice
	Zol	Active K+/P2X7 receptor/ROS pathway	NLRP3↑, caspase-1↑, IL-1*β*↑	Impair oral socket wound healing	db/db mice
	BSP	Inhibit NLRP3 inflammasome activation	TNF-*α*↓, ROS↓, IL-1*β*↓, IL-18↓, caspase-1↓, NLRP3↓	Accelerate diabetic wound healing, suppress macrophage infiltration, and promote angiogenesis	High fat-diet feeding combined with streptozocin in C57BL/6 mice; macrophages
	YVAD	The caspase-1 inhibitor	IL-1*β*↓, IL-18↓, NLRP3↓	Improve wound healing	Keratinocytes and Mp
	Glyburide	Close ATP-sensitive potassium channels	IL-1*β*↓, IL-18↓, NLRP3↓	Improve wound healing	Keratinocytes and Mp
	Melatonin	Inhibition of NLRP3 inflammasome activation	TNF-*α*↓, IL-1*β*↓, IL-6↓, IL-8↓, NLRP3↓	Promote diabetic wound healing	Keratinocytes
	MFG-E8	Intergrin *β*3-limited P2X7 receptor pathways	Active caspase-1↓, IL-1*β*↓, IL-18↓, NLRP3↓	Improve angiogenesis and accelerates wound healing	Mfge8-/-diabetic mice, Mfge8-/-neutrophils
	Fenofibrate	Inhibition of ROS/TXNIP/NLRP3 pathway	TXNIP↓, active caspase-1↓, IL-1*β*↓ ,IL-18↓, NLRP3↓	Accelerate wound healing	STZ-induced diabetic mice, EPC
	NAC	A free radical scavenger	ROS↓, IL-1*β*↓ ,IL-18↓, caspase-1↓, NLRP3↓	Improve the wound healing	STZ-induced diabetic mice

Abbreviation: GC-AgNPs-CGP: gallocatechin-silver nanoparticle-impregnated cotton gauze patches; Nrf2: nuclear factor erythroid-2-related factor; HO-1: heme oxygenase 1; TLR4: Toll-like receptor 4; NF-*κ*B: nuclear factor kappa-B; MDA: malonaldehyde; SOD: superoxide dismutase; Nqo-1:NADPH quinone oxidoreductase-1; Keap-1: Kelch-like ECH-associated protein 1; MMP-2: matrix metalloproteinase-2; NLRP3: Nod-like receptor protein 3; ASC: apoptosis-associated speck-like protein containing; IL-1*β*: interleukin-1; STZ: streptozocin; TNF-*α*: tumor necrosis factor-alpha; IL-6: interleukin-6; GPx: glutathione peroxidases; MMP-9: matrix metalloproteinase-9; AMPK: adenosine monophosphate-activated protein kinase; mTOR: mechanistic target of rapamycin; IL-10: interleukin-10; LC3A/B: microtubule-associated protein 1A/1B-light chain 3; TLR4: Toll-like receptor 4; CXCR2: C-X-C motif chemokine receptor 2; Mp: macrophage; VEGF: vascular endothelial growth factor; TGF-*β*1: transforming growth factor beta1; MMP10: matrix metalloproteinase-10; CXCL12: chemokine C-X-C motif ligand 12; ROS: reactive oxygen species; IL-18: interleukin-18; P2X7: BBG: brilliant blue G; Zol: zoledronate; BSP: Bletilla striata polysaccharide; YVAD: Tyr-Val-Ala-Asp; MFG-E8: milk fat globule epidermal growth factor VIII; NAC: N-acetylcysteine; TXNIP: thioredoxin-interacting protein.

## Data Availability

All data included in this study are available upon request by contact with the corresponding author.
